# Contrast sensitivity and subjective visual disturbances across the psychosis continuum

**DOI:** 10.1080/13546805.2026.2678602

**Published:** 2026-05-26

**Authors:** Yunyang Zhang, Jan W. Brascamp, Jacqueline Y. Bao, William Quackenbush, Christophe Delay, Sarah Jones, Nathan T. Carter, Steven M. Silverstein, Brian P. Keane, Katharine N. Thakkar

**Affiliations:** a Department of Psychology, Michigan State University, East Lansing, MI, USA; b Department of Psychology and Neuroscience, Duke University, Durham, NC, USA; c Department of Neuroscience, Vanderbilt University, Nashville, TN, USA; d Department of Psychiatry, University of Rochester Medical Center, Rochester, NY, USA

**Keywords:** Schizophrenia, visual disturbances, contrast sensitivity, clinically high risk

## Abstract

**Introduction:**

Subjective visual disturbances and objective visual deficits are common across the psychosis spectrum. Few investigations have tested them in the same sample to ask whether subjective disturbances may arise from low-level visual processing deficits. This study assessed whether reduced contrast sensitivity (CS), a low-level visual processing deficit in people with schizophrenia (PSZ), may contribute to subjective visual disturbances across the psychosis spectrum.

**Methods:**

PSZ (*n* = 54) and controls (*n* = 54) matched for gender, age, and race/ethnicity, together with High PLE (*n* = 55) and Low PLE (*n* = 45) matched for gender, age, race/ethnicity, education, and IQ completed a CS task and the Bonn Scale, a structured interview assessing visual distortions. Group differences in CS and Bonn scores as well as their correlations with social risk factors were evaluated. In addition, the Bonn Scale’s factor structure was examined.

**Results:**

PSZ and High PLE endorsed more distortions than controls. In PSZ, distortion severity, but not CS, correlated with social risk factors and clinical symptoms. Only PSZ showed reduced CS, and no associations between distortions and CS were observed.

**Conclusions:**

Findings do not support a bottom-up account of visual distortions; instead, they may arise from higher-order processes.

## Introduction

Individuals on the schizophrenia spectrum show altered visual perception, as evidenced by subjective symptoms (e.g. visual hallucinations and distortions) as well as altered performance on objective, laboratory-based measures of visual performance (Adámek et al., 2022). Examining vision is particularly informative because, unlike higher-order cognitive or social processes, the visual system is both well-characterized at neural and computational levels and closely linked to clinical symptoms. This makes the visual system ideal for uncovering how specific neural disturbances give rise to perceptual abnormalities and for identifying early markers of psychosis risk.

Visual hallucinations occur in approximately 27% of individuals with a schizophrenia spectrum disorder (Waters et al., 2014) and refer to visual perception in the absence of corresponding sensory input. Subjective visual disturbances include distortions in features such as colour, brightness, motion, shape, or size (Silverstein & Lai, 2021). Visual disturbances are milder but more common than visual hallucinations (Silverstein & Lai, 2021), occurring in approximately 60% of individuals with schizophrenia (Schwarzer et al., 2022; Silverstein, 2016). Visual disturbances are part of a broader constellation of basic symptoms (Gross et al., 1987) – a set of subclinical experiential changes that unfold subtly during the prodromal period (Gross & Huber, 2010; Klosterkötter et al., 2001). Visual disturbances in particular are markers of psychosis risk and prognostic indicators of a psychotic disorder diagnosis: they are observed among individuals at clinical high risk for psychosis (Kimhy et al., 2007; Klosterkötter et al., 2001) as early as childhood (Schubert et al., 2005), and predict the later development of psychosis (Klosterkötter et al., 2001). Visual disturbances have other important clinical correlates: they are associated with greater clinical symptoms (Keane et al., 2018), earlier age of onset (Keane et al., 2018), greater depressive symptoms, lower quality of life (Schwarzer et al., 2022), and suicidal ideation (Granö et al., 2015). Finally, recent studies indicate overlapping risk factors between visual disturbances and psychosis (Keane et al., 2018), including poorer premorbid social functioning. Combined, these findings suggest clinical relevance of visual disturbances, their potential utility as early warning signs, and partly shared etiological mechanisms with psychosis.

A largely separate body of literature has reported objective visual processing deficits in individuals with schizophrenia using laboratory-based psychophysical paradigms (McCleery et al., 2020; Silverstein, 2016; Zemon et al., 2021). One of the most well-documented visual processing deficits in individuals with an established schizophrenia diagnosis is reduced contrast sensitivity (Butler et al., 2008; Kéri et al., 2002; Linares et al., 2025; Zemon et al., 2021), which refers to a reduced ability to detect differences in light and dark (i.e. between high and low luminance values) and distinguish an object from its background. Contrast sensitivity emerges from computations in an early visual processing hierarchy from retina to the lateral geniculate nucleus of the thalamus to primary visual cortex (Shapley & Lennie, 1985). At low spatial frequencies, contrast sensitivity depends more on the magnocellular (M) pathway – a fast, luminance-driven system specialised for coarse detail and motion – whereas contrast sensitivity for high spatial frequency stimuli depend more on the parvocellular (P) pathway, which is slower, colour-sensitive, and optimised for fine detail and high spatial resolution (Butler et al., 2008; Merigan & Maunsell, 1993). Reduced contrast sensitivity has been related to impairments in working memory, perceptual organisation (Herrera et al., 2021), reading (Martínez et al., 2013), and social and emotional outcomes (Butler et al., 2009; Keane et al., 2014) in people with schizophrenia. Evidence also suggests contrast sensitivity varies across illness stages: while reduced contrast is often observed in chronic patients, enhancements have been reported in unmedicated first episode patients (Kelemen et al., 2013; Kiss et al., 2010) though findings are inconsistent (Kadivar et al., 2024). Whether contrast sensitivity is altered prior to the onset of illness is unclear. To our knowledge, only two studies have examined individuals at clinical high-risk for psychosis, and results are mixed (Kadivar et al., 2024; Kéri & Benedek, 2007).

It has been theorised that abnormal processing of input at early stages of the visual hierarchy manifests as a noisy feedforward signal that contributes to the development of an incorrect model of the external world by higher order brain regions, which manifests in the form of subjective distortions and misinterpretations about reality (Adámek et al., 2022). Were this the case, one would expect a relationship between low-level objective visual alterations (e.g. contrast sensitivity deficits) and subjective visual distortions. A few studies have reported such a link in people with schizophrenia. However, this evidence is limited, and the pattern of findings is inconsistent. For example, Kéri et al. (2005) found that reduced contrast sensitivity using a method that purportedly biases towards the M pathway (Skottun & Skoyles, 2008) was associated with more anomalous perceptual experiences among people with chronic schizophrenia, whereas Kiss et al. (2010) observed the opposite relationships among first-episode patients. More recently, Ifrah et al. (2024) found spatial frequency-specific associations, such that greater perceptual disturbances were related to enhanced contrast sensitivity at low spatial frequencies but reduced sensitivity at the mid frequencies. These discrepancies may partly reflect the potentially separable perceptual anomalies being collapsed into a single measure. Identifying more fine-grained clusters of subjective visual distortions may be necessary to clarify their mechanisms. However, no study has yet examined whether such clusters exist or whether specific distortions show differential relationships with objective visual alterations.

The current study aimed to clarify the relationship between subjective visual distortions and contrast sensitivity across the psychosis spectrum. First, we examined group differences in contrast sensitivity and subjective visual disturbances in individuals ranging from those experiencing distressing psychotic-like experiences (PLEs) but not necessarily seeking treatment, to individuals with established psychotic illness. If impaired contrast sensitivity contributes to subjective visual distortions, then High PLEs should show poorer contrast sensitivity and greater subjective visual disturbances than their control group. Moreover, group differences in contrast sensitivity should be at least as large as those observed for subjective visual distortions. Second, we examined, among PSZ and the High PLE group, the associations of subjective and objective visual anomalies with social risk factors and clinical symptoms. Perceived discrimination and life adversity were selected because they are strongly associated with psychosis risk (Heinz et al., 2013), and these stressors have been hypothesised to impact neural processes contributing to perceptual abnormalities (Varchmin et al., 2021). If low-level visual impairments contribute to visual distortions, subjective and objective alterations should show similar patterns of associations with clinical and social variables, consistent with shared etiology and clinical impact. Third, we assessed the relationship between contrast sensitivity and visual impairments across groups to test whether objective and subjective visual anomalies covary in a manner consistent with shared mechanisms. Finally, we explored whether distinct clusters of visual distortions could be identified.

## Methods and materials

### Participants

This study was approved by the Michigan State University Institutional Review Board. All participants gave written informed consent and were compensated. Exclusion criteria for all participants included: 1) prior head injury with loss of consciousness over an hour, 2) history of neurological disorder, 3) prior ECT within the past 3 months, 4) moderate or severe substance use disorder within the past six months, 5) Premorbid IQ < 70 as measured by the Wechsler Test for Adult Reading (WTAR) (Wechsler, 2001), and 6) corrected visual acuity worse than 20/50 using the Sloan-letter near vision chart assessed at 16 inches. Testing limits were between 20/20 and 20/200.

### Individuals with schizophrenia or schizoaffective disorder (PSZ) and healthy controls (HC)

54 individuals with schizophrenia or schizoaffective disorder (PSZ) and 54 healthy controls (HC) were recruited from outpatient mental health facilities and community advertisements (Table 1). Diagnoses were based on an electronic version of the Structured Clinical Interview for *DSM-5* Axis I disorders (Brodey et al., 2016), medical records, and collateral informants. HC were additionally excluded for any personal history of any DSM-5 Axis I disorders or first-degree relatives with schizophrenia spectrum or bipolar disorders. After excluding 9 PSZ and 10 HC for poor task performance (see Supplementary Methods), 44 eligible HC and 45 PSZ remained. Comparison of included and excluded PSZ and HC is presented in Supplementary Table 1. Eligible groups were matched for sex, gender, age, race, and ethnicity; however, IQ and years of education were significantly higher in HC. Despite significant differences, we did not include IQ and education as covariates, since they are likely tied to the illness process itself and thus reflect meaningful disorder-related variance. 33 PSZ were taking antipsychotic medication, and chlorpromazine (CPZ) equivalent dosages were calculated.

### Psychotic-like experiences (PLE) groups

100 university students endorsing a high (High PLE) or low (Low PLE) level of distressing psychotic-like experiences (55 High PLE and 45 Low PLE) were recruited from the Michigan State University psychology subject pool (Table 2). PLE status was determined using the Prodromal Questionnaire-Brief (Loewy et al., 2011). Individuals endorsing more than 8 items with a distress rating of 4 or 5 were categorised into the High PLE group, consistent with published screening guidelines in non-help-seeking samples (Savill et al., 2018). Cutoff for the Low PLE group was a maximum of one endorsed item with a distress score of one. Exclusion criteria for High and Low PLE participants included current use of psychotropic medication and DSM-5 Axis I diagnosis of schizophrenia, schizoaffective disorder, schizophreniform disorder, or bipolar disorder. Low PLE individuals were further excluded for any current Axis I diagnosis or a first-degree relative with a schizophrenia spectrum or bipolar disorder diagnosis. 4 High PLE and 2 Low PLE participants were excluded based on performance, leaving 43 Low PLE and 51 High PLE participants. Groups were matched for age, sex, gender, race, ethnicity, IQ, education, and visual acuity.

### Clinical, psychosis risk and social risk factor assessments

Clinical symptoms were assessed in PSZ using the Brief Psychiatric Rating Scale (BPRS) (Lukoff et al., 1986), Scale for the Assessment of Positive Symptoms (SAPS) (Andreasen, 1984), and Scale for the Assessment of Negative Symptoms (SANS) (Andreasen, 1989). Severity of distressing PLEs was assessed in the High PLE group using the PQB Weighted Distress Score (Savill et al., 2018). Current and lifetime subjective visual distortions were assessed across groups using the 17 visual items from the Bonn Scale for the Assessment of Basic Symptoms (BSABS) interview (Gross et al., 1987). Each item received a score of 1 (present), 0.5 (possible), or 0 (absent) and was scored by two trained research staff with high interrater reliability (ICC = .997). Due to the restricted range of scores for current visual distortions, we used the lifetime score for all analyses. History of discrimination and cumulative adversity were assessed in all groups using lifetime scores on the Perceived Discrimination Scale (Williams et al., 1997) and the Life Events Checklist (Weathers et al., 2013), respectively.

### Contrast sensitivity paradigm and procedure

**Figure 1.**
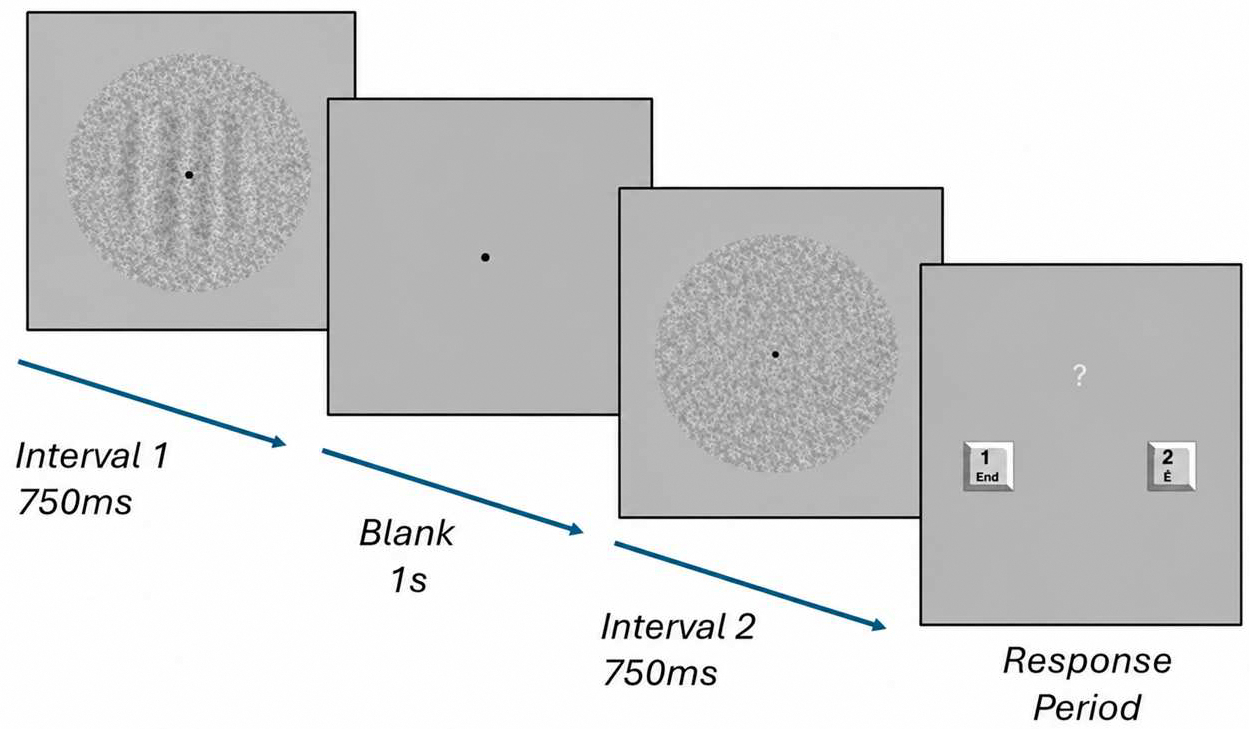
Contrast sensitivity paradigm.

Full paradigm details are described in Supplementary Methods. Briefly, contrast sensitivity was measured using a two-interval forced choice paradigm as illustrated in Figure 1. Each trial included two 750 ms intervals separated by a 1 s inter-stimulus interval. On each of the two intervals, participants were presented with a visual noise patch (approximately 3 degrees of visual angle); overlaid on one of those noise patches was a vertical grating with a spatial frequency of 2 cycles/degree and at a contrast selected randomly from 6 Michelson contrast values (0.005, 0.007, 0.010, 0.014, 0.020, and 0.029). Participants were instructed to select which of the two intervals contained in the grating by pressing either 1 or 2 on the keyboard. The experiment comprised 80 trials and lasted approximately 5 min.

### Data analysis

#### Quantifying contrast sensitivity

Contrast sensitivity was measured by estimating the minimum contrast level needed to reliably detect the grating. We calculated at each contrast level the proportion of correct answers. For each participant, the relationship between the proportion of correct responses and log contrast level was fitted with vertically scaled and shifted cumulative Gaussian functions with three parameters: mean, standard deviation, and “lapse rate”: the proportion of trials in which the participant makes a response error regardless of the clarity of their perception. Mean corresponds to threshold. While standard deviation is closely related to slope, it is not the same because slope also depends on lapse rate. A lapse rate of 0 indicates that the function spans the full range between the chance accuracy (50%) and perfect accuracy (100%); otherwise, its vertical range is scaled down. Standard deviation indicates slope for a given lapse rate, so lapse rate and standard deviation together determine slope. Contrast threshold (i.e. fitted mean parameter) corresponds to the natural-log contrast value that yields 75% correct performance. Contrast sensitivity, in turn, was computed as the negative of contrast threshold. To ensure data quality, we applied performance exclusion criteria detailed in Supplementary Methods.

### Statistical analyses

Statistical analyses examined group differences in subjective and objective visual anomalies and probed their relationships with each other as well as with clinical variables and social risk factors. All statistical analyses were conducted using IBM SPSS Statistics version 28.0.1.1(IBM, Armonk, NY).

Because the BSABS probes a range of visual anomalies – from flashing lights to distorted faces – we first conducted an item factor analysis to evaluate the dimensionality and reliability of this measure in PSZ (see Supplementary Methods). This analysis supplemented the current sample of 51 PSZ with data from 43 PSZ from a previously published study conducted at Rutgers, The State University of New Jersey (Keane et al., 2018). Demographics of the combined sample of 94 individuals are presented in Supplementary Table 2.

Group differences (i.e. PSZ vs HC; High PLEs vs Low PLEs) in subjective visual disturbances (total and potential subscale scores) were evaluated using independent sample t-tests. For contrast sensitivity differences between HC and PSZ, we assessed the effects of visual acuity and age as covariates on contrast sensitivity using separate linear regression models, given findings that visual acuity plays a role in explaining how contrast sensitivity changes with spatial frequencies (Bi et al., 2023), and that aging significantly affects contrast sensitivity (Zhuang et al., 2021). For High versus Low PLEs, given the tight age range and equivalent visual acuity, we used an independent samples t-test to assess group difference in contrast sensitivity. In separate analyses, group differences in lapse rate from the cumulative Gaussian function were analyzed to evaluate the potential contribution of attentional or other non-sensory contributions. All equal variance assumptions were retained unless Levene’s test indicated otherwise.

We assessed the relationships between subjective and objective visual abnormalities, clinical symptoms, and social risk factors among PSZ and High PLE groups using Spearman’s rho. Given the distribution of responses on the BSBAS within the High PLE group (See Supplementary Figure 1), we categorised participants as with or without visual distortions and performed independent t-tests to examine BSBAS group differences in contrast sensitivity, clinical symptoms, and social risk factors.

Additionally, Bayes factors (BF_01_) were computed for PLE comparisons to quantify the strength of evidence for null or alternative hypothesis and were interpreted using conventional benchmarks: 0.1–0.33 (moderate for H_1_); 0.33–1(anecdotal for H_1_), 1(no preference), 1–3 (anecdotal for H_0_), and 3–10 (moderate for H_0_) (Lee & Wagenmakers, 2014).

## Results

### Contrast sensitivity

#### PSZ versus HC

**Figure 2.**
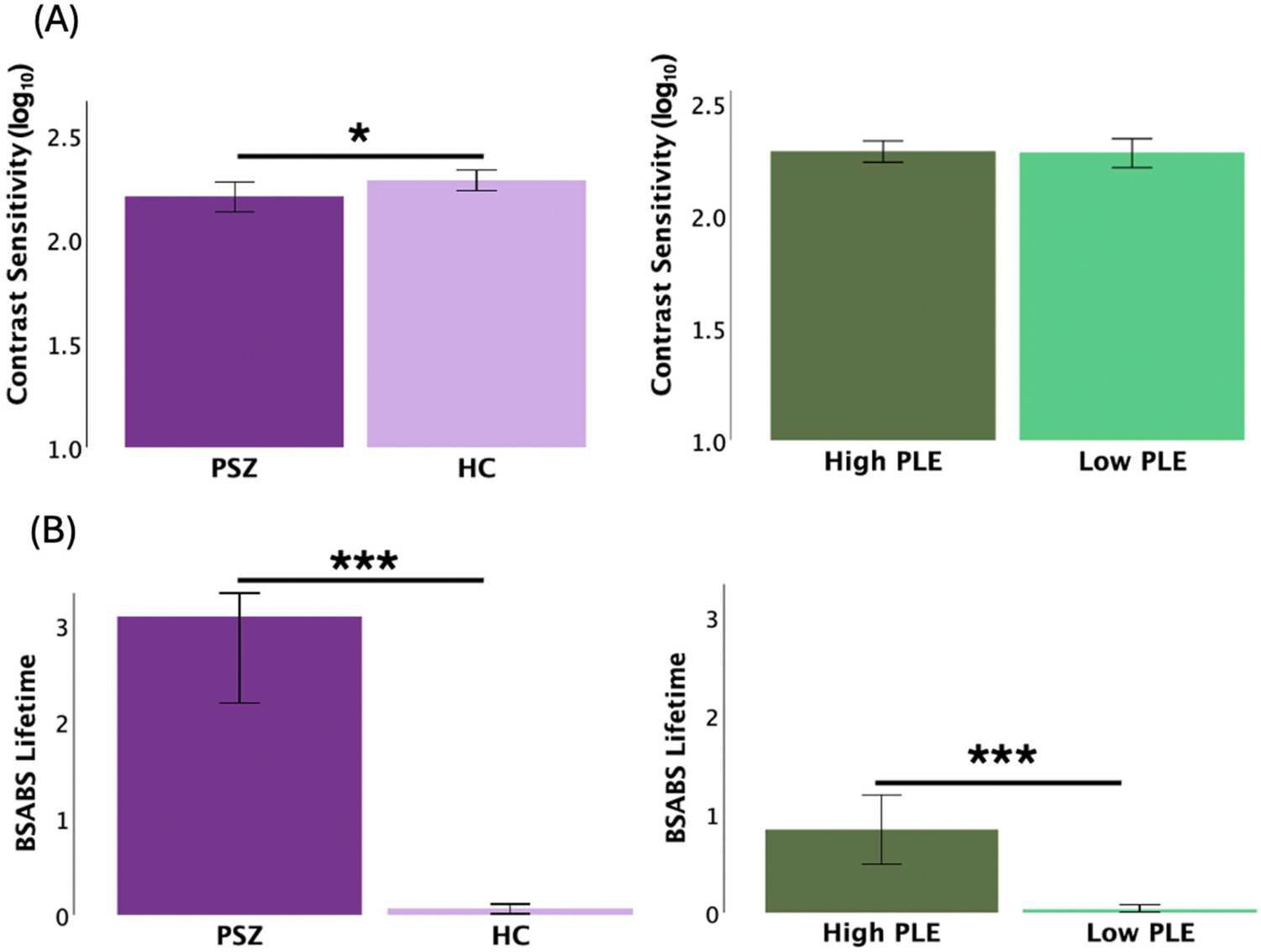
Differences in (A) contrast sensitivity and (B) subjective visual distortions between HC and PSZ (left) and High and Low PLE (right). (***p < .001, *p < .05).

First, simple linear regressions showed that age, t(87) = −2.44, β = −0.25, *p* = .02, but not visual acuity, t(86) = −.71, β = −0.08, *p* = .48, significantly predicted contrast sensitivity such that older individuals showed reduced contrast sensitivity. To confirm that visual acuity did not contribute meaningfully to model fit, we compared two multiple regressions: one with group, age, and visual acuity as predictors, R^2^ = .09, and one with only group and age as predictors, R^2^ = .11, further supporting the exclusion of visual acuity. The final multiple regression model including group and age showed a significant main effect of group, t(86) = −2.11, *p* = .04, β = −0.22, such that PSZ showed reduced contrast sensitivity relative to HC when controlling for the effects of age (Figure 2A, left). Importantly, there was no difference in lapse rates between PSZ and HC, t(78.59) = 1.45, *p* = .15, d = .31, and adding lapse rate to the model predicting contrast sensitivity from group and age resulted in a minimal change in the standardised beta value for group and a near overlap in confidence interval for the estimates (see Supplementary Results). This result argues against general cognitive or motivational factors explaining group differences in contrast sensitivity.

### High PLE versus low PLE

In contrast to findings in PSZ, no significant differences in contrast sensitivity were observed between Low and High PLE participants (Figure 2A, right; t(92) = 0.17, *p* = .86, *d* = .04). Consistent with frequentist results, the Bayesian independent-samples t-test provided moderate evidence in favour of the null hypothesis, supporting the absence of group differences (BF_01_ = 6.21). There was no group difference in lapse rate, t(90) = 0.49, *p* = .63, *d* = .10.

### Subjective visual distortions

Item factor analysis supported a single-factor solution, justifying the use of the total score (See Supplementary Results). Both PSZ and High PLE endorsed more subjective visual distortions than the corresponding comparison group (PSZ vs HC: t(41.26) = 6.88, *p* < .001, *d* = 1.51; High vs Low PLE: t(50.05) = 4.58, *p* < .001, *d* = .88; Figure 2B).

### Relationships among contrast sensitivity, visual distortions, clinical measures and social risk factors in PSZ and high PLE

Correlation matrices are shown in Table 3. In PSZ, more lifetime visual distortions were associated with higher scores on the SAPS, *r*_*s*_ = 0.58, *p* < .001, and BPRS, *r*_*s*_ = 0.57, *p* < .001, but not SANS, *r*_*s*_ = −0.02, *p* = 0.9 (See Supplementary Results for correlations with SAPS subscales). In addition, greater visual distortions were associated with social risk factors: higher levels of lifetime perceived discrimination, *r*_*s*_ *=* 0.46, *p* = .002, and greater cumulative adversity, *r*_*s*_ *=* 0.37, *p* = .017. There were no significant correlations between contrast sensitivity and clinical/social factors or CPZ equivalent dosages, all *r*_*s*_’s < 0.40; *p* > 0.1. To determine if the correlations with symptoms were significantly different for subjective (BSABS) versus objective (contrast sensitivity) measures, we used Steiger’s z-test for dependent correlations within each group with contrast sensitivity as the referent correlation. Results indicated that correlations with BSABS were significantly stronger than those with contrast sensitivity for BPRS, Z = −3.73, *p* < .001, SAPS, Z = −2.65, *p* = .01, and perceived discrimination, Z = −2.45, *p* = .01. In the High PLE group, we did not observe any significant differences between those with and without visual distortions on social risk factors or PQB distress scores, nor any relationship between contrast sensitivity and clinical and social measures (all *p*’s > 0.06).

In contrast to prior findings, we did not observe a significant relationship between contrast sensitivity and subjective visual distortions in PSZ, *r*_*s*_ = 0.03, *p* = .84; However, there was a statistical trend for High PLEs endorsing visual distortions (N = 22) to have poorer contrast sensitivity than those that did not (N = 27), though this difference was not significant, t(47) = − 1.94, *p* = .06, *d* = − .56. Given the moderate effect size, we conducted a Bayesian independent-samples t-test, yielding BF_01_ = 0.96, indicating inconclusive evidence for either the null or alternative hypothesis. Formal correction for multiple comparisons was not applied, as our primary aim was to compare patterns of associations using dependent correlation tests, rather than to evaluate individual correlations. Therefore, the results should be cautiously interpreted given the number of statistical comparisons.

## Discussion

The main aim of this study was to bridge findings of alterations in fundamental aspects of visual perception, indexed by contrast sensitivity, and subjective visual disturbances in individuals on the psychosis spectrum. While some studies suggest that changes in basic visual perception may contribute to subjective disturbances or share an etiology, our findings largely do not support this. First, although subjective visual disturbances were observed in both PSZ and High PLEs, only PSZ showed altered contrast sensitivity. Second, contrast sensitivity and visual distortions showed differing patterns of relationships with clinical and social correlates. Finally, no significant relationship was found between contrast sensitivity and subjective visual experiences. Here, we relate these findings to previous studies, consider interpretations, highlight limitations, and speculate upon clinical relevance.

PSZ had weakened contrast sensitivity compared to HC – a finding that could not be explained by age-related decline (Elliott et al., 1990; Zhuang et al., 2021). The effect size of the difference, however, was smaller than in prior studies; this may be explained by the generalizability of the patient sample. First, groups did not differ in lapse rate, contrary to a robust body of evidence indicating attentional problems in PSZ, suggesting that this sample of PSZ may be higher functioning than previous samples. Although, our metric of inattention (lapse rate) should be interpreted cautiously as these parameters are likely noisy estimates of true lapse rate (Wichmann & Hill, 2001). Second, performance criteria disproportionately excluded PSZ with the most severe symptoms. Combined, these factors limit sample generalizability in a way that would lead to underestimation of contrast sensitivity reductions in PSZ. However, group differences remained after controlling for age. These findings suggest that general inattention, guessing, or age-related decline cannot account for reduced contrast sensitivity in PSZ.

We also examined contrast sensitivity in young adults with significant and distressing PLEs. According to clinical staging models of psychosis (Cross et al., 2014), the High PLE group falls in Stage 1a-1b, wherein PLEs are considered an early marker of vulnerability. We did not observe a group difference in contrast sensitivity between the High and Low PLE groups, underscoring inconsistent findings across the psychosis spectrum. Of the two existing studies in clinical high-risk samples, contrast sensitivity was reduced in one and enhanced in the other relative to community controls. Notably, these two studies used different paradigms: Kadivar et al. (2024) probed near-threshold detection, while Kéri and Benedek (2007) used a paradigm that examines contrast gain control mechanisms. Furthermore, Harper et al. (2020) reported that participants with high schizotypal traits from a broad undergraduate sample showed reduced contrast sensitivity but only at a low spatial frequency (although this interaction between group and spatial frequency may be confounded (Bi et al., 2023)). Variable findings are likely accounted for by methodological differences across studies and indicate unmodeled moderators. In sum, while our findings suggest that altered contrast sensitivity may be a more proximal illness marker rather than a marker of risk, more studies are needed to clarify its expression in the psychosis risk state.

We observed robust subjective visual disturbances in both PSZ and High PLE groups compared to their respective controls, aligning with prior studies suggesting that these distortions may serve as early markers of psychosis vulnerability (Klosterkötter et al., 2001; Schwarzer et al., 2022). The finding of visual disturbances in both groups, alongside contrast sensitivity deficits only among PSZ, challenges the hypothesis that contrast sensitivity impairments contribute to subjective visual distortions. Were that hypothesis true, we should expect to find equivalent contrast sensitivity impairments in PSZ and High PLE groups, concomitant with subjective distortion differences. Further challenging this hypothesis are the different patterns of social and clinical correlates of contrast sensitivity and visual distortions. In PSZ, greater subjective visual disturbances were associated with more severe positive symptoms, aligning with Keane et al. (2018), and general psychiatric symptoms. Their links with cumulative adversity and perceived discrimination suggest the role of social-environmental stressors. One proposed pathway in Varchmin et al. (2021) is that exposure to threatening social situations heightens attention to external cues, increasing the likelihood of attributing salience to irrelevant stimuli and thereby contributing to perceptual distortions. We did not observe any social or clinical correlations with contrast sensitivity in either group. Together, such differential correlations with social risk factors for contrast sensitivity and subjective visual distortions further challenge accounts of subjective visual distortions driven by contrast sensitivity abnormalities. However, other explanations should be considered. For example, it is possible that an inability to adapt to or compensate for reduced contrast sensitivity at higher levels of a visual hierarchy may relate to clinical and social variables.

As a final finding challenging the hypothesis that contrast sensitivity impairment drive subjective distortions, contrast sensitivity was not significantly related to visual distortions in PSZ, and evidence in High PLEs was inconclusive. This pattern suggests that visual distortions captured by the BSABS may reflect higher-order disruptions arising from integrative visual processes, rather than or in addition to early mechanisms like contrast detection. This interpretation is consistent with our item factor analysis that yielded a single factor, and aligns with Kéri et al. (2005), who found that perceptual organisation, involving higher-order visual integration, was the best predictor of BSABS scores.

Another possibility is that the link between contrast sensitivity and visual distortions varies by illness stage or stimulus features. For example, Kiss et al. (2010) found associations in first-episode patients only under M-pathway-biased conditions, and Ifrah et al. (2024) observed spatial-frequency-specific patterns in a clinically high-risk group: greater anomalous experiences related to better sensitivity at low frequencies but poorer sensitivity at mid-range frequencies. Together, these findings suggest that subjective visual anomalies may arise from multiple mechanistic pathways rather than a single low-level deficit.

Several limitations should be noted. First, our experimental design was underpowered to detect small or medium effects, particularly for within-group correlation analyses. Second, we cannot parcel out antipsychotic medication effects, which may reduce contrast sensitivity in PSZ (Chen et al., 2003; Kéri et al., 2002). Medication cannot, however, explain greater subjective distortions along the full schizophrenia spectrum as unmedicated High PLEs also had more severe visual disturbances. Third, using a single spatial frequency did not allow us to examine M- or P-pathway-specific alterations. Fourth, most High PLE participants in the current study are unlikely to transition to psychosis, limiting interpretations about the prodromal period. Fifth, performance criteria disproportionately excluded older HC and PSZ with the most severe symptoms. This systematic exclusion bias may limit the generalizability of group comparisons and correlations. Sixth, our measure of visual acuity did not extend beyond 20/20, which may have blunted its observed effect on contract sensitivity. Lastly, we cannot evaluate whether adaptive or compensatory mechanisms for contrast sensitivity impairments at a higher level of processing may complicate associations between contrast sensitivity and visual distortions, symptoms, and social risk factors (Foxe et al., 2005; Silverstein et al., 2020; Silverstein & Keane, 2011).

Limitations notwithstanding, our findings do not support the hypothesis that low-level visual deficits, as assessed by contrast sensitivity, contribute to visual distortions through a bottom-up mechanism. Instead, the presence of visual distortions across the psychosis spectrum – including among individuals at clinical high risk, where contrast sensitivity remains largely intact – suggests that these experiences may be shaped more by higher-order processes than early sensory deficits. By jointly assessing subjective visual distortions and low-level visual processing in PSZ and High PLE, these findings help clarify how visual changes unfold across illness stages. Future work could extend these findings using a range of spatial frequencies or by sampling individuals further along the illness trajectory. Finally, the absence of alterations in contrast sensitivity in individuals at risk for psychosis carries potential clinical implications: if confirmed in larger longitudinal samples, subjective visual distortions may be a more proximal and clinically accessible indicator of emerging psychopathology than low-level visual measures, although more work is needed before drawing firm conclusions about their utility in risk prediction.

## Supplementary Material

Supp 1

Supplemental data for this article can be accessed online at https://doi.org/10.1080/13546805.2026.2678602.

## Figures and Tables

**Table 1.** Demographic characteristics of the patient and control groups.

	HC (*n* = 44) Mean (s.d.)	PSZ (*n* = 45) Mean (s.d.)	Statistic	*p*-value

Age	37.3(10.8)	35.2(11.3)	*t* = 0.93	.36
Sex (M/F)	24/20	29/16	*X*^2^ = .91	.34
Gender (M/F/O)	27/17/0	31/13/1	*X*^2^ = 1.80	.41
IQ	109.8(7.8)	105.7(9.3)	*t* = 2.23	.03
Race^a^ (Black, White, Other)	8/26/10	8/31/5	*X*^2^ = 3.09	.38
Ethnicity (Hispanic, non-Hispanic)	3/41	4/40	*X*^2^ = 0.16	.69
Education (yrs)	17.1(3.3)	14.0(2.1)	*t* = 5.31	<.001
Visual acuity (logMAR)^b^	0.21(0.10)	0.27(0.11)	*t* = -2.33	.02
Lifetime Perceived Discrimination	1.6(1.9)	2.4(2.9)	*t* = −1.44	.16
Cumulative Adversity	6.7(4.8)	11.1(5.5)	*t* = −3.89	<.001
Years of Illness		14.1(11.2)		
CPZ Equivalent		322.9(349.7)		
BPRS		40.3(8.8)		
SAPS		18.7(18.7)		
SANS		25.6(15.6)		

a Other category includes: 1 Native American/Alaska Native, 5 Asian/Indian, 5 Multiracial, and 2 Not Listed.

b 20/20 corresponds to LogMar of zero and more negative values are better.

**Table 2.** Demographic characteristics of the High PLEs and Low PLEs.

	Low PLEs (*n* = 43) Mean (s.d.)	High PLEs (*n* = 51) Mean (s.d.)	Statistic	*p*-value

Age	19.6(1.3)	19.6(1.6)	*t* = −0.21	.83
Sex (M/F)	23/20	21/30	*X*^2^ = 1.42	.23
Gender (M/F/O)	22/21/0	22/29/0	*X*^2^ = 0.60	.44
IQ	107.6(7.4)	109.0(6.9)	*t* = −0.99	.33
Race (Black, White, Other)	2/28/13	7/26/18	*X*^2^ = 3.00	.22
Ethnicity (Hispanic, non-Hispanic)	1/42	3/48	*X*^2^ = 0.72	.40
Education (yrs)	13.2(1.0)	13.4(1.3)	*t* = −0.86	.39
Visual Acuity (logMAR) ^a^	0.23(0.93)	0.22(0.95)	*t* = 0.81	.42
Lifetime Perceived Discrimination	0.5(0.8)	1.3(1.3)	*t* = −3.57	<.001
Cumulative Adversity	2.9(2.4)	7.0(3.5)	*t* = −6.55	<.001
PQB Total Score^b^	0.1(0.3)	13.4(2.6)	*t* = −36.66	<.001
PQB Weighted Distress Score^b^	0.1(0.3)	51.0(8.7)	*t* = −41.75	<.001

a 20/20 corresponds to LogMar of zero and more negative values are better

b Total Score Refers to the number of symptom items a person endorsed; Weighted distress score was calculated as the sum of the distress levels experienced for each endorsed item.

**Table 3.** Correlation matrix between contrast sensitivity, visual distortions, clinical symptoms and social-risk factors among PSZ (A) and High PLEs (B)

(A) PSZ						

Variable	Cumulative Adversity	Contrast Sensitivity	BSABS Lifetime	BPRS	SANS	SAPS

Contrast sensitivity	0.003					
BSABS Lifetime	0.370*	0.033				
BPRS	0.229	−0.196	0.566***			
SANS	−0.157	−0.216	−0.020	0.274		
SAPS	0.301	0.055	0.578***	0.764**	0.011	
Lifetime perceived discrimination	0.372*	−0.050	0.464**	0.167	0.023	0.168

(B) High PLE						

Variable	Cumulative Adversity	Contrast Sensitivity	BSABS Lifetime	PQB Distress		

Contrast sensitivity	0.057					
BSABS Lifetime	0.187	−0.239				
PQB Distress	0.139	−0.241	0.208			
Lifetime perceived discrimination	0.392**	0.018	0.281	0.224		

(***p < .001, *p < .05).

## Data Availability

Data will be made available upon request to the corresponding author.
