# Supplementary material for: Contrast sensitivity and subjective visual disturbances across the psychosis continuum
Source: Cogn Neuropsychiatry. Author manuscript; Available in PMC 2026 Jul 8. (PMC13345213; doi:10.1080/13546805.2026.2678602)
Supplement: Supp 1 [file NIHMS2185021-supplement-Supp_1.docx]

**Supplemental Methods**

***Contrast sensitivity paradigm and procedure***

Contrast sensitivity was measured using a two-alternative forced choice paradigm. Each trial included two 750 ms intervals separated by a 1 s inter-stimulus interval during which only a fixation point was shown. On each of the two intervals, participants were presented with a visual noise patch (approximately 3 degrees of visual angle); overlaid on one of those noise patches was a vertical grating. The grating was constructed using a luminance value that varied sinusoidally across space with a spatial frequency of 2 cycles/degree and with a contrast that varied per trial. Possible Michelson contrasts of the sine wave were 0.005, 0.007, 0.010, 0.014, 0.020, and 0.029. The noise patch was constructed by giving each pixel in the stimulus a random luminance drawn from a uniform distribution ranging from 0.95 times the background luminance to 1.05 times the background luminance. For the stimulus that did contain the grating, the grating’s luminance values were added pixel-by-pixel to the noise patch luminance. For all stimuli, with or without grating, the final step toward computing pixel luminance values was to multiply the resulting luminance values (relative to the background luminance), with a Gaussian gain with a standard deviation of 0.7 degrees. Regarding the size of the stimulus, the stimulus does not have a hard edge but rather fades away as a result of this multiplication with a Gaussian gain. If we assume that one stops seeing the stimulus at 2 standard deviations away from the center then the diameter is 4 standard deviations, which is about 3 degrees.

Before the task, the participants received training on the task. They first completed a number of easy practice trials (Michelson contrast of the grating set to 0.05). These were repeated until 5 consecutive responses were correct. After that, they continued to harder practice trials (Michelson contrast of 0.029). Those were repeated until 3 consecutive responses were correct, after which the real experiment started. Participants were given a maximum of 11 attempts for easy practice trials and 9 attempts for harder practice trials. If the total number of attempts exceeded those limits, then the experiment was terminated with a message on the screen telling the experimenter that the participant did not reach the criterion. The experimenter would then discuss the task with the participant and attempt to identify any misunderstandings or difficulties, after which the participant would be allowed to try the experiment (including practice) again. After a second round of practice trials, all participants reached the required performance criteria to move on to the main experiment.

***Performance exclusion criteria***

Participants' performance was assessed by fitting a psychometric curve that plotted the proportion of correct responses as a function of log contrast. For a participant's data to be included, the curve was required to meet two criteria. First, the slope had to be sufficiently steep, defined as increase in accuracy of at least 0.28 within a log contrast range of 1.5 units, centered on the threshold contrast level (defined as the point yielding 75% correct performance). This criterion ensured that the proportion of correct responses rose substantially as contrast level increased, as expected if a participant understood the task and could detect the grating at least at the highest contrasts used. The second criterion was that the sum of squared error distance between the fitted curve and the raw data needed to be below 0.025, ensuring exclusion for improper curve fit.

***Item factor analysis of the Bonn Scale for the Assessment of Basic Symptom (BSABS) in PSZ***

To examine the factor structure and psychometric properties of the BSABS visual subsection, we utilized item factor analysis (IFA). The sample of participants included in this analysis comprised 51 PSZ from current study and 43 participants from Rutgers University (participant details are provided in Keane et al. (2018)). Demographics for the combined participant sample are presented in **Supplementary Table 2.**

To conduct the IFA, we used a graded response model (GRM) (Samejima, 1969). The GRM assumes item responses are ordered and estimates two types of parameters for each item. First, each item has a discrimination parameter, *a*, which is conceptually similar to a factor loading; in the case of multiple latent factors, each item has as many discrimination parameters as there are factors. Higher values of *a* for an item indicate greater sensitivity to differences in the underlying trait. Next, a set of threshold parameters are estimated, which in the context of ordinal data, directly corresponds to the trait level at which an individual is most likely to respond using option *k* + 1 rather than choosing option *k*. For example, in a three-option measure, if *b*_1_ = -1.0, then those with a trait level higher than -1.0 *SD* away from the mean are more likely to respond “Neutral” rather than “Disagree,” and if *b*_2_ = +1.50, those with an attribute level higher than 1.50 are more likely to respond “Agree” rather than “Neutral;” regardless of the number of factors, each item has one less threshold parameter than the number of response options. A notable strength of IFA models is their ability to assess reliability across levels of the latent factor, allowing us to examine whether an item or scale score is more or less reliable at different levels of the attribute.

**Supplementary Results**

***Role of lapse rate in group difference in contrast sensitivity***

To further evaluate role of lapse rate in contrast sensitivity between HC and PSZ, we include lapse rate as a covariate into the model predicting contrast sensitivity from age and group. In the model including age, group and lapse rate, the standardized coefficient for group was β = -0.196, 95% CI [-0.168,0.004]. In comparison, the model including only age and group yielded the standardized coefficient for group β = -0.216, 95% CI [-0.175, -0.005]. The two confidence intervals overlapped substantially, and the magnitude of the group effect changed minimally with the inclusion of lapse rate.

***Item factor analysis of BSABS***

First, we estimated a single-factor model, and then a two-factor model. Examining model fit estimates, the single-factor model showed similar fit and more desirable statistical properties than the two-factor model despite fewer parameters estimated. Specifically, the unidimensional model showed good absolute fit to the data, *M*_2_(103)=128.32, *p* = .045, RMSEA=.051 (95% CI: .007; .078), SRMR=.099, CFI=.96, TLI=.97, whereas the two-factor model showed negligibly-better fit, *M*_2_(87)=92.83, *p* = .315, RMSEA=.027 (95% CI: .000; .064), SRMR=.081, CFI=.99, TLI=.99, with the CFI and TLI of the two-factor model indicating overfitting, showing values very close to 1.0. Review of the two-factor model revealed that item parameter estimates fell outside the expected range of values for some items, further indicating over-fitting. In contrast, in the single-factor model, discrimination parameter estimates showed a reasonable range – from 1.06 to 3.04 – values that indicate items were reliable indicators of the latent factor. Item parameters for the unidimensional model are shown in **Supplementary Table 3**.

**Supplementary Figure 2** plots the reliability of the measure as a function of attribute level on the 0 to 1 scale typical of other measures of reliability (e.g., coefficient alpha). As can be seen, the test is most reliable for attribute levels above the mean of zero. Scores in the current sample ranged from -.98 to +2.32, and the BSABS showed reliability greater than .80 for 75% of the current sample.

***Correlations between visual distortions (BSABS) and positive symptoms subscales in PSZ***

In PSZ, greater lifetime visual distortions were associated with higher SAPS scores *r_s_*=0.58, *p*<.001. The SAPS overall scores were calculated as the sum of four subscales in accordance with Andreasen (1984): Hallucination, Delusion, Bizarre Behavior and Positive Formal Thought Disorder. Visual distortions were associated with more severe hallucinations, *r_s_*=0.63, *p*<.001, and delusions *r_s_*=0.47, *p*=.002; associations with bizarre behavior, *r_s_*=0.20, *p*=.21, and positive formal thought disorder, *r_s_*=-0.14, *p*=.39, were not significant. Steiger’s z-test for dependent correlations indicated that visual distortions were more strongly associated with hallucinations than with bizarre behavior (Z = 2.41, p = .008) and positive formal thought disorder (Z = 3.94, p < .001). Associations between visual distortions and with delusions were stronger than with positive formal thought disorder (Z = 2.80, p = .003) but did not differ significantly from associations with bizarre behavior (Z = 1.52, p = .06). These findings indicated that the association between visual distortions and SAPS in the patient group is primarily driven by hallucinations and delusions.

**Supplementary Table 1.** Demographic and clinical measures of individuals with schizophrenia and healthy controls that were excluded or included based on their performance on contrast sensitivity**.**

|  | HC included (n=44) | HC excluded  (n=10) | Cohen’s d | PSZ included (n=45) | PSZ excluded (n=9) | Cohen’s d |
| --- | --- | --- | --- | --- | --- | --- |
|  | Mean (s.d) | Mean (s.d) |  | Mean (s.d) | Mean (s.d) |  |
| **Age** | 37.3 (10.8) | 46.4 (13.7) | -0.80 | 35.2 (11.3) | 34.9 (10.8) | 0.02 |
| **Sex (M/F)** | 24/20 | 6/4 |  | 29/16 | 8/1 |  |
| **Gender (M/F/O)** | 27/17/0 | 7/3/0 |  | 31/13/1 | 8/1/0 |  |
| **IQ** | 109.8 (7.8) | 107.2 (10.0) | 0.31 | 105.7 (9.3) | 108.6 (9.7) | -0.31 |
| **Race**  **(Black, White, Other)** | 8/26/10 | 5/4/1 |  | 8/31/5 | 1/8/0 |  |
| **Ethnicity**  **(Hispanic, non-Hispanic)** | 3/41 | 0/10 |  | 4/40 | 1/8 |  |
| **Education (yrs)** | 17.1 (3.3) | 16.0 (2.7) | 0.35 | 14.0 (2.1) | 13.6 (1.5) | 0.19 |
| **Visual acuity (logMAR)** ^a^ | 0.2 (0.1) | 0.3 (0.1) | -0.45 | 0.3 (0.1) | 0.3 (0.1) | -0.47 |
| **Lifetime Perceived Discrimination** | 1.6 (1.9) | 2.3 (2.1) | -0.31 | 2.4 (2.9) | 2.1 (3.0) | 0.10 |
| **Cumulative Adversity** | 6.7 (4.8) | 5.9 (3.4) | 0.18 | 11.1(5.5) | 8.5 (5.1) | 0.47 |
| **BPRS** |  |  |  | 40.3 (8.8) | 47.1(13.2) | -0.70 |
| **SANS** |  |  |  | 25.6 (15.6) | 31.9 (18.6) | -0.39 |
| **SAPS** |  |  |  | 18.7 (18.7) | 32.4 (29.1) | -0.67 |
| **CPZ equivalence** |  |  |  | 322.9 (349.7) | 696.7 (521.1) | -1.00 |

^a^ 20/20 corresponds to LogMar of zero and more negative values are better.

**Supplementary Table 2.** Demographic variables of combined participant sample recruited from The State University of New Jersey and the current study.

|  | Participants (n=94)  Mean (s.d) |
| --- | --- |
| **SZ/ Unspecified psychotic disorder** | 82/12 |
| **Age** | 34.0(11.3) |
| **Gender (M/F/O)** | 66/27/1 |
| **Race**  **(Black, White, Other)** | 25/48/20 |
| **Education (yrs)** | 13.7(2.3) |
| **Illness Duration** | 13.0(11.9) |
| **CPZ equivalence** | 401.3(366.5) |
| **SAPS** | 20.7(20.1) |
| **BSABS Total** | 2.7(3.2) |

**Supplementary Table 3.** Reliability of the measure at different levels of the measured attribute

| Item | *a* | *b*_1_ | *b*_2_ |
| --- | --- | --- | --- |
| Blurred/unclear vision | 1.38 | .78 | 1.13 |
| Transitory blindness-temporary blindness affecting whole visual field | 2.73 | 1.43 | 1.50 |
| Partial seeing – only parts of object are perceived | 1.82 | 1.89 | 1.99 |
| Hypersensitivity to light or certain optic stimuli | 1.56 | 1.04 | 1.48 |
| Photopsias – seeing flashes or stars | 1.07 | .68 | .89 |
| Porropsia – objects seem to be closer or farther away but unchanged in their size | 1.68 | 1.71 | 1.88 |
| Micropsia – objects appear smaller than they are.  Macropsia – objects appear bigger than they are | 2.03 | 1.56 | 1.71 |
| Metamorphopsia – also called dysmorphopsia, when straight lines or objects appear wavy or deformed | 1.17 | 1.92 | 2.21 |
| Metachromopsia – changes in color vision | 2.95 | .94 | 1.47 |
| Changes in the perception of the face/body of others | 1.97 | 1.21 | 1.38 |
| Changes in the perception of own face or body | 2.19 | 1.11 | 1.32 |
| Pseudomovement of objects | 2.12 | .81 | .98 |
| Double vision – double, oblique, slanting(sloping) and reversed vision | 1.71 | .95 | 1.25 |
| Disturbances of the estimation of distances | 1.42 | 1.82 | 1.99 |
| Disintegration of the linearity of (objective) contours of objects | 1.93 | 1.59 | 1.67 |
| Dysmegalopsia – inability to judge object sizes accurately | 3.04 | 2.14 | . |
| Persistence of stimuli – abnormally long-lasting optic stimuli and sebsequent vision of things seen in reality minutes, hours or days before respectively | 2.18 | 1.54 | 1.69 |

**Supplementary Figure 1.** Distribution of responses on the BSABS in the High PLE group


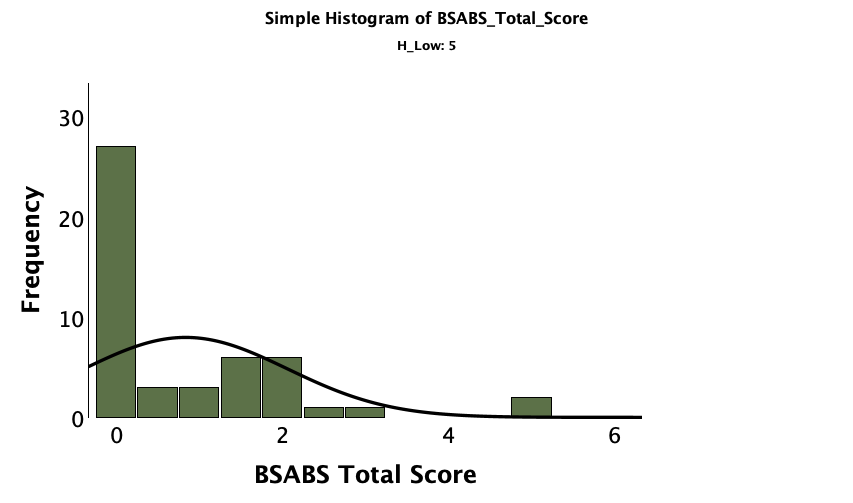


**Supplementary Figure 2.** Reliability of the measure at different levels of the measured attribute

**References**

Andreasen, N. C. (1984). Scale for the assessment of positive symptoms. *Group*, *17*(2), 173-180.

Keane, B. P., Cruz, L. N., Paterno, D., & Silverstein, S. M. (2018). Self-Reported Visual Perceptual Abnormalities Are Strongly Associated with Core Clinical Features in Psychotic Disorders [Original Research]. *Frontiers in Psychiatry*, *Volume 9 - 2018*.

Samejima, F. (1969). Estimation of latent ability using a response pattern of graded scores. *Psychometrika Monograph Supplement*, *34*(4, Pt. 2), 100-100.
